# Arthroscopic Assisted Distal Radial Ulnar Joint Reconstruction Using a Modified Anatomic Ligament Reconstruction

**DOI:** 10.1016/j.eats.2025.103926

**Published:** 2025-11-29

**Authors:** Julianne Gillis, Spencer B. Chambers, Anna L. Gorsky, Nina Suh, Michael B. Gottschalk, Eric R. Wagner

**Affiliations:** a Department of Orthopedics, Division of Hand and Upper Extremity Surgery, Emory University, Atlanta, Georgia, U.S.A.; b Department of Plastic & Reconstructive Surgery, Hand & Upper Limb Center, Western University, London, Ontario, Canada

## Abstract

Distal radioulnar joint (DRUJ) instability is a complex pathology that typically occurs due to injury of the triangular fibrocartilage complex. In the acute setting, repair is often successful. However, in the chronic setting or after a prior failed repair, reconstructive methods aim to improve stability using an extrinsic radioulnar soft tissue link. Traditional surgical techniques are technically demanding and require a broad exposure, compromising extrinsic stabilizers. Modifications to these methods using arthroscopy facilitate preservation of ulnocarpal and DRUJ secondary stabilizers, while providing excellent visualization for anatomic passage of the graft and placement of the foveal bone tunnel. We describe a simple and effective arthroscopic reconstruction method for restoring and reinforcing the native anatomy of the critical stabilizers of the DRUJ.

The distal radioulnar joint (DRUJ) is critical for forearm rotation and stability, relying heavily on soft tissue constraints due to limited bony support.^1^, ^2^, ^3^ The triangular fibrocartilage complex (TFCC) is the primary stabilizer, and pathology arising from traumatic or degenerative conditions compromises DRUJ function.^4^, ^5^, ^6^ While repair of acute TFCC tears is feasible, chronic injuries, failed repairs, or poor tissue quality require reconstruction.

The anatomic ligament reconstruction described by Adams and Berger,^7^^,^^8^ using a palmaris longus (PL) tendon through extra-articular radial and ulnar bone tunnels, is the gold standard but requires extensive exposure and disrupts secondary stabilizers with limited intra-articular visualization. Arthroscopy has evolved from diagnosis to reconstruction,^9^ enabling preservation of extrinsic stabilizers and improved precision.^8^^,^^10^^,^^11^ Irreparable TFCC tears resulting in DRUJ instability remain a challenging pathology without an ideal treatment protocol. This arthroscopic modification of the anatomic ligament reconstructive procedure^7^ is a valuable tool in the treatment algorithm for irreparable injuries (Fig 1).

**Fig 1:**
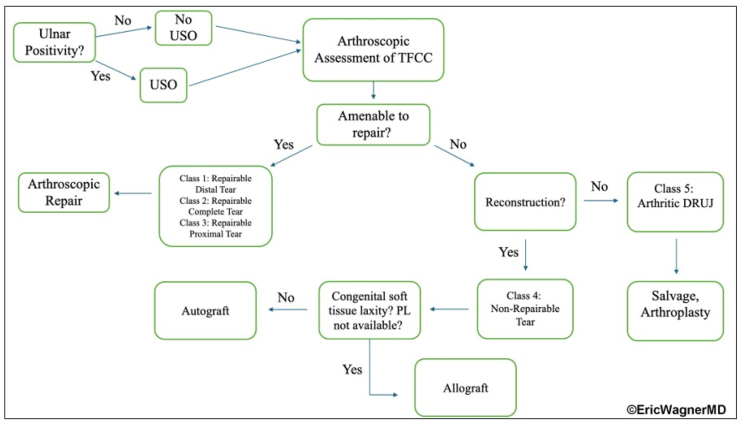
Treatment algorithm for irreparable triangular fibrocartilage complex (TFCC) tears. Diagram illustrating the decision-making pathway for managing irreparable TFCC tears with distal radioulnar joint (DRUJ) instability. Prior to assessing the TFCC, ulnar positivity will dictate the need for ulnar shortening osteotomy. If the TFCC is not amenable to repair, reconstruction is indicated unless the patient has an arthritic DRUJ that is only compatible with salvage procedures.

## Surgical Technique

### Position, Marking, and Portals

**Fig 2:**
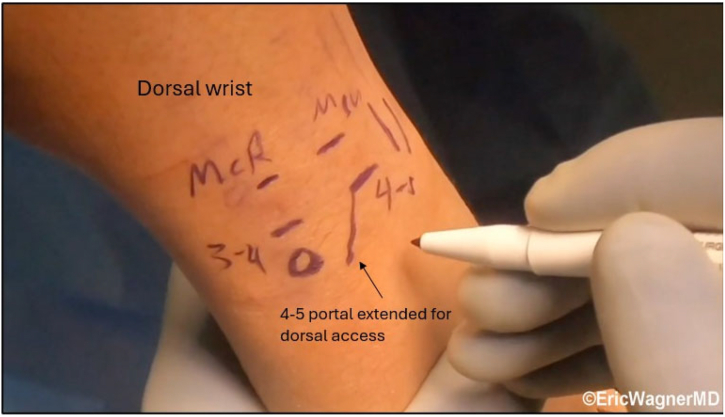
Arthroscopic portals for distal radioulnar joint (DRUJ) reconstruction. Illustration of dorsal wrist anatomy highlighting the radiocarpal 3-4, 4-5, and ulnar volar portals used in arthroscopic-assisted DRUJ reconstruction. The 4-5 portal is extended proximally and radially for dorsal access, while the ulnar volar portal facilitates graft passage, preserving ulnocarpal and DRUJ secondary stabilizers. The patient is positioned supine in a wrist traction tower, and this is the right wrist.

This outpatient procedure utilizes regional anesthesia. Standard portals include radiocarpal, 3-4, and 4-5, with the 4-5 extended proximally and radially, as well as an ulnar volar portal for graft passage (Fig 2).

### TFCC Reconstruction

**Fig 3:**
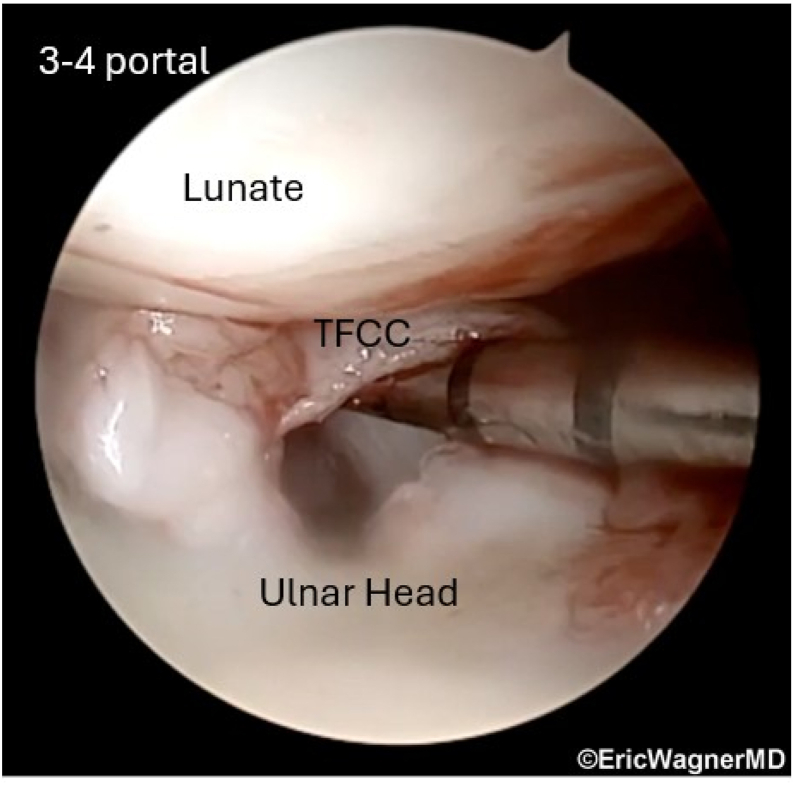
Positive hook test. The hook test assesses the peripheral triangular fibrocartilage complex (TFCC) integrity. Viewing through the 3-4 portal, a probe is applying radial traction and lifting the TFCC off the ulnar head, demonstrating the TFCC tear. The patient is positioned supine in a wrist traction tower, and this is the right wrist.

**Fig 4:**
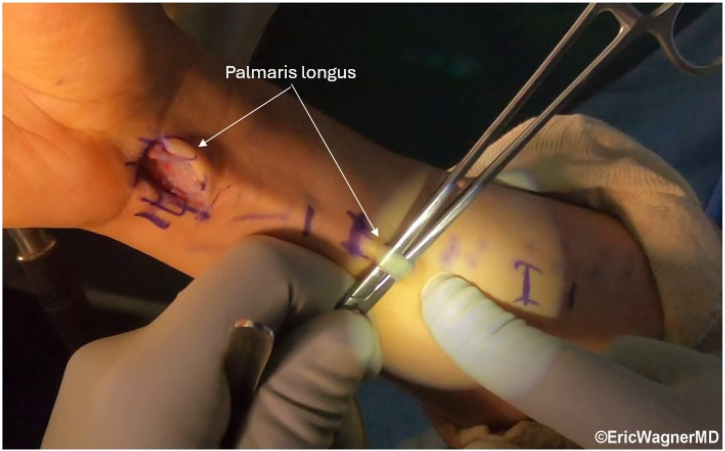
Palmaris longus graft harvest. Harvest of the palmaris longus tendon through 3 transverse incisions over the volar forearm. The tendon, at least 15 cm in length, is prepared with a whipstitch using a 2-0 nonabsorbable suture to enhance structural integrity and aid graft passage. The patient is positioned supine in a wrist traction tower, and this is the right wrist.

After confirming an irreparable TFCC tear with diagnostic arthroscopy (3-4, 4-5 portals) (Fig 3, Video 1), synovectomy and debridement are performed. The PL is then harvested using 3 volar incisions, yielding ≥15 cm (Fig 4). The length of the graft is whipstitched, using 2-0 nonabsorbable suture coming out each end to assist with structural integrity and graft passage. A technical tip is to start two-thirds of the way from each end, crossing over each other by one-third in the middle of the graft.

**Fig 5:**
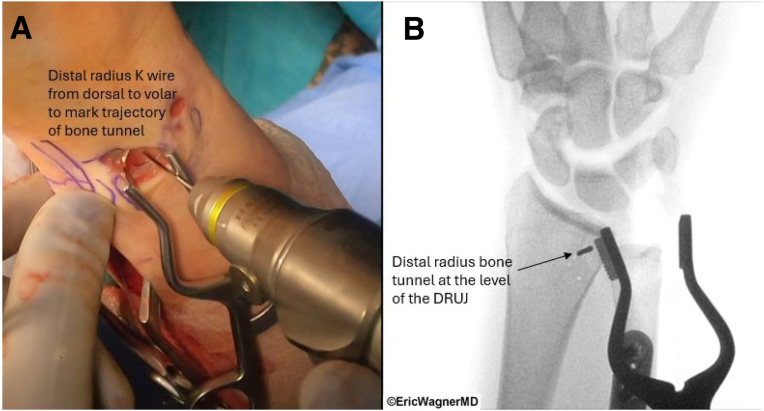
Distal radius bone tunnel creation. (A) K-wire placement from dorsal to volar at the level of the distal radioulnar joint, approximately 10 mm proximal and radial from the radius edge. Cannulated drills (2.5 mm, then 3.5 mm) create the bone tunnel for palmaris longus graft passage, guided by direct visualization. (B) Fluoroscopic image confirming the K-wire placement of the distal radius bone tunnel prior to the cannulated drills. The patient is positioned supine in a wrist traction tower, and this is the right wrist.

**Fig 6:**
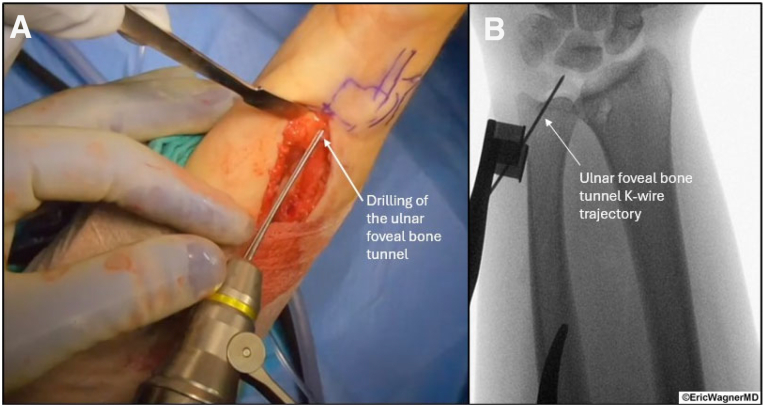
Ulnar foveal bone tunnel creation. (A) K-wire placement from the ulnar diaphysis to the ulnar fovea under direct visualization. Placement is also confirmed via arthroscopic visualization of the ulnar head through the 3-4 portal. (B) Fluoroscopy confirms the trajectory, followed by cannulated drilling (2.5 mm, then 3.5 mm) to create the ulnar bone tunnel. The patient is positioned supine in a wrist traction tower, and this is the left wrist.

Next, small incisions are created to expose the volar and dorsal distal radius. The volar-ulnar aspect of the distal radius is exposed by extending the distal PL incision proximally just radial to the flexor carpi ulnaris and developing the interval between the carpal tunnel contents and ulnar neurovascular structures. Similarly, the dorsal ulnar aspect of the radius is exposed by extending the 4-5 portal incision proximally and radially, utilizing an interval between the fourth and fifth extensor compartments. Using fluoroscopy, K-wires are passed from dorsal to volar at the level of the DRUJ, approximately 10 mm proximal and radial from the edge of the radius (Fig 5). Cannulated drills are used to create a 2.5-mm and then ultimately a 3.5-mm bone tunnel. The ulnar bone tunnel is created using a subcutaneous incision along the ulnar border. With the ulnar head visualized through the 3-4 portal, a TFCC Guide (Arthrex) through the 4-5 portal is used to place a guidewire from the ulnar diaphysis to the fovea (Fig 6). With trajectory confirmed with fluoroscopy, the wire is stabilized with a Mosquito, and subsequent cannulated drills are used, starting at a 2.5-mm and eventually a 3.5-mm bone tunnel. Using this bone tunnel, if there is any TFCC tissue, a 22-gauge needle loaded with a 2-0 polydioxanone suture is passed through the remnant TFCC in 2 different locations (Table 1). Polydioxanone is used as a shuttle stitch to pass a 2-0 nonabsorbable suture through the graft reconstruction sutures.

**Table 1:** Pearls and Pitfalls

Pearls	Pitfalls
Utilize a 22-gauge needle loaded with a 2-0 polydioxanone suture as a passing mechanism to enhance precision in suture placement. Ensure precise placement of the distal radius tunnel at the volar ulnar corner and the ulnar tunnel to create a robust bone bridge, minimizing fracture risk. Start with a 2.5-mm drill for bone tunnels, then expand to 3.5 mm to facilitate smooth graft passage and reduce the risk of bone fracture. Use the palmaris longus harvest site as the volar radius incision to reduce the number of incisions and associated morbidity. Pass the triangular fibrocartilage complex repair stitch before the graft to optimize visualization and ensure effective repair. Bring both graft limbs through the 6R portal and position them in the graft passer before guiding them through the ulnar tunnel for streamlined passage. Release wrist traction during graft tightening to achieve a more anatomic reduction, then immobilize the wrist in a neutral position to maintain stability. Employ 2 separate bone anchors to enhance reduction stability and provide redundancy in case of anchor failure.	Using an excessively thick graft can complicate passage through bone tunnels, increasing the risk of intraoperative challenges. Failure to center the graft passer on the ulna may lead to difficulties in graft passage through the tunnel, compromising reconstruction. The fifth extensor compartment may obstruct the 6R portal; ensure the portal is directed beneath this compartment to avoid tendon damage. Using interference screws can lead to osteolysis around bone tunnels, increasing the risk of graft failure or fracture.

**Fig 7:**
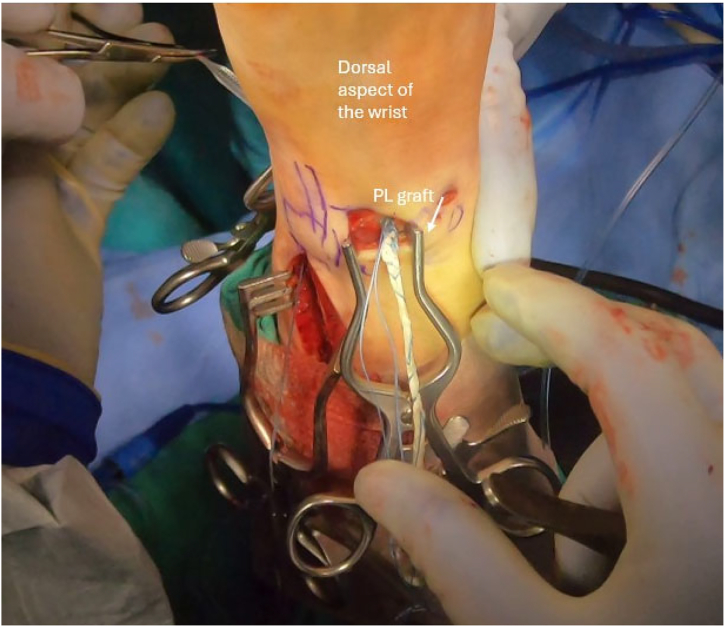
Palmaris longus graft passage. Intraoperative photograph illustrating the palmaris longus graft passed dorsal to volar through the distal radius bone tunnel. A grasper retrieves the volar limb between the short radiolunate and volar ulnolunate ligaments, guiding it through the ulnocarpal joint to the 4-5 portal. The patient is positioned supine in a wrist traction tower, and this is the left wrist.

**Fig 8:**
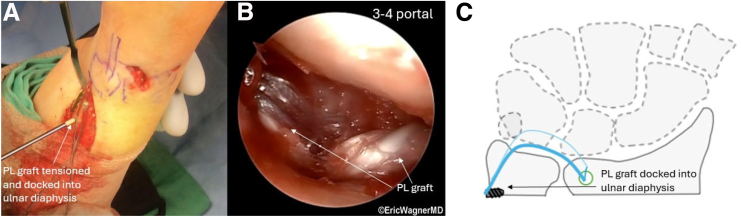
Graft tensioning and anchoring. (A) The graft is tensioned in neutral forearm rotation and docked into the ulnar diaphysis using 2 bone anchors, incorporating triangular fibrocartilage complex remnant sutures if viable. (B) Arthroscopic image of the final graft construct, with volar and dorsal limbs of the palmaris longus graft shuttled through the ulnar bone tunnel. (C) Illustration of the overall graft construct. The green circle represents the radial bone tunnel, the thick blue line is the dorsal palmaris longus graft limb, the thin blue line is the volar palmaris longus graft limb, and the gray screw represents the anchors at the ulnar diaphysis securing the graft limbs in place. The patient is positioned supine in a wrist traction tower, and this is the left wrist.

To begin the graft passage, the PL is loaded into a graft passer (Arthrex) (Fig 7), which is passed from dorsal to the volar through the distal radius tunnel. Viewing through the 3-4 portal and working through the 4-5 portal, a grasper is passed between the short radiolunate and volar ulnolunate ligaments out the volar incision to retrieve the volar limb of the PL graft and draw it through the ulnocarpal joint and out of the 4-5 portal. The dorsal limb is passed beneath extensor tendons, placing both ends of the PL graft at the 4-5 portal. A shuttling suture is passed up the ulnar bone tunnel and retrieved through the 4-5 portal. A graft passer is drawn back to the joint, where it is loaded with the volar and dorsal limbs of the PL to be positioned within the ulnar bone tunnel. The graft ends are both shuttled through the ulnar bone tunnel. The graft is tensioned maximally in neutral forearm rotation, docking the previously placed whipstitches into the ulnar diaphysis using 2 bone anchors (Fig 8). The TFCC repair stitches are also placed in the anchors if possible.

### Postoperative Management

Patients are immobilized in a sugar tong splint for 14 days, transitioning to a Muenster splint in neutral rotation for 6 weeks. Active/passive motion begins, progressing to strengthening at 12 weeks, with full activity resumed at 16 weeks.

## Discussion

The DRUJ is a complex diarthrodial joint that allows for smooth forearm rotation and load transfer. With limited bony constraints, stability depends heavily on soft tissue.^12^ DRUJ disruption can lead to instability, pain, and loss of motion, resulting in limited function and pain. Irreparable TFCC injuries are particularly challenging and can occur in the setting of poor tissue quality, failed primary repairs, high-energy trauma, large irreparable tears, and congenital insufficency.^13^ Tears are typically acquired through trauma or degeneration, whereas insufficiency is observed in conditions of soft tissue laxity, such as Ehlers-Danlos syndrome. Although imaging may diagnose tears, diagnostic wrist arthroscopy is the primary method for visualizing the location and assessing the severity.

**Fig 9:**
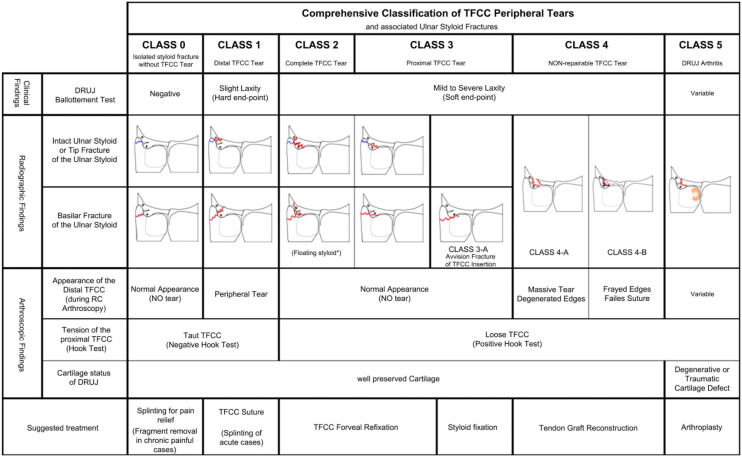
Atzei classification of triangular fibrocartilage complex (TFCC) tears. Schematic diagram of the Atzei classification^6^ for TFCC tears, focusing on Class 4 (irreparable) injuries. Class 4A (massive tears with degenerated edges) and Class 4B (frayed edges or failed repairs) are depicted, highlighting indications for arthroscopic reconstruction due to poor tissue quality or large defects.

According to the Atzei classification^6^ (Fig 9), Class 4 injuries represent irreparable TFCC tears requiring tendon graft reconstruction. Class 4A involves a massive tear with degenerated edges, and Class 4B involves a TFCC with frayed edges or prior failed suture repair. The main difference is the reason for irreparability: 4A has a large defect, while 4B has poor healing capacity. Both present with TFCC laxity, a soft endpoint, and a positive hook test, and they are indicated for reconstruction.^13^ Arthroscopic assessment of tissue quality is helpful, determining if the tissue is strong enough for a primary repair. Treatment options for irreparable TFCC tears include open versus arthroscopic graft reconstruction. The traditional anatomic ligament reconstruction described by Adams and Berger^7^^,^^8^ can be successful but is associated with inherent deficiencies,^8^ including challenges involving poor visualization,^14^ significant dorsal extrinsic ligament soft tissue disruption,^15^ and potentially a more difficult recovery.^16^, ^17^, ^18^, ^19^, ^20^ Arthroscopy improves upon these shortcomings, allowing for an anatomic reconstruction by precisely passing the graft between the short radiolunate and ulnolunate ligaments, identifying the foveal attachment for graft passage, preserving the ulnocarpal and remnant DRUJ extrinsic ligaments, and protecting the associated blood supply (Table 2).^10^^,^^21^ The minimally invasive nature of arthroscopy, with smaller incisions and less surgical dissection, also potentially promotes a quicker recovery of forearm range of motion due to less scarring (Table 2).^22^^,^^23^

**Table 2:** Advantages and Disadvantages

Advantages	Disadvantages
Arthroscopy provides clear, direct visualization of intra-articular pathology, enabling precise evaluation of injury severity before proceeding with surgical intervention. Facilitates identification and concurrent treatment of associated injuries, including potential triangular fibrocartilage complex repairs when necessary. Allows accurate graft placement between the short radiolunate and ulnolunate ligaments, ensuring anatomic reconstruction. Enables exact identification of the foveal attachment for optimal graft passage, enhancing DRUJ stability. Minimizes disruption to ulnocarpal and remnant DRUJ extrinsic ligaments while preserving the associated blood supply, promoting better healing. Decreases overall soft tissue disruption, potentially leading to less scar tissue formation and improved recovery. Utilizes bone anchors in the ulna for secure, low-profile fixation, reducing the likelihood of hardware prominence.	Arthroscopic DRUJ reconstruction demands advanced wrist arthroscopy expertise, which may result in longer operative times, particularly during the learning curve. Requires specialized arthroscopic equipment, elevating procedural costs and necessitating trained support staff for proper handling and maintenance. Arthroscopic exposure and tower setup can complicate intraoperative fluoroscopy, potentially affecting surgical precision.

DRUJ, distal radioulnar joint.

Although technically demanding, arthroscopic-assisted DRUJ reconstruction offers a safe and effective alternative to conventional open reconstruction, with the added benefits of potentially faster recovery from minimally invasive incisions. Studies have shown that arthroscopic techniques yield better functional outcomes when compared to open repairs.^9^^,^^24^^,^^25^ Tse et al.^10^ reported a 133% improvement in pronation and a 116% improvement in supination with arthroscopy, compared to 90% and 87% improvements, respectively, with open surgery. Additionally, arthroscopy has been shown to enhance grip strength by preserving ulnocarpal stability at the extensor carpi ulnaris sheath and surrounding tissues.^26^

**Fig 10:**
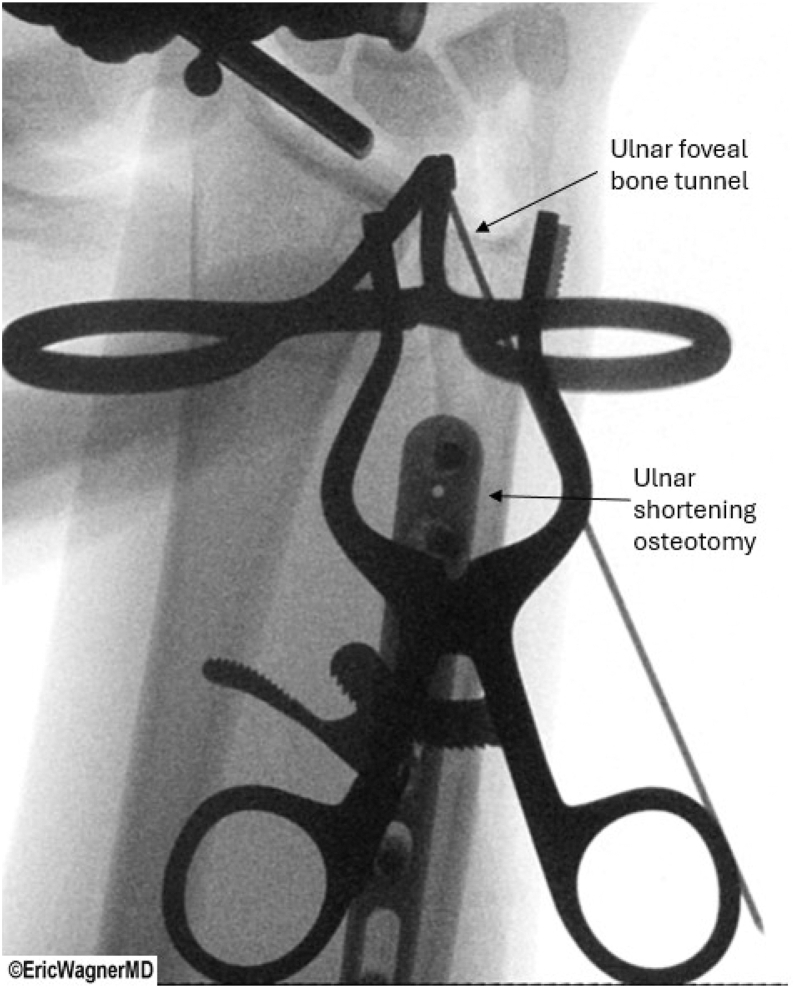
Concomitant ulnar shortening osteotomy. Intraoperative X-ray of a patient who underwent ulnar shortening osteotomy performed with triangular fibrocartilage complex reconstruction in biomechanically disadvantaged distal radioulnar joints (DRUJs) (e.g., positive ulnar variance). The procedure tightens ulnocarpal ligaments and offloads the reconstruction, showing the osteotomy site and fixation to restore DRUJ congruity. The patient is positioned supine in a wrist traction tower, and this is the right wrist.

Additional considerations for this intervention include patients with connective tissue disorders or inherently biomechanically disadvantaged DRUJs. In patients with poor tissue quality, the use of PL or a split-hamstring allograft is prudent to minimize the risk of secondary soft tissue laxity of the repair.^27^ The success of allografts has been demonstrated in similar procedures.^27^^,^^28^ In other cases, the DRUJ is biomechanically disadvantaged due to the ulna being longer than the radius. In these cases, concomitant reconstruction with ulnar shortening osteotomy can address all aspects of this pathology, as it offloads the reconstruction while also tensioning the ulnocarpal ligaments (Fig 10). Advantages of adding a ulnar shortening osteotomy to TFCC reconstruction include tightening the extrinsic ulnocarpal ligaments^29^ and avoiding further damage to the distal articular surface of the ulna. As this procedure comes with complications, such as nonunion or incongruity of the distal radioulnar joint, preoperative counseling is critical.^30^

Irreparable TFCC tears present a complex surgical challenge with many considerations. This arthroscopic approach, tailored to patient anatomy, improves function and has been seen to reduce traditional complications, advancing the surgical management of irreparable tears.

## Disclosures

The authors declare the following financial interests/personal relationships which may be considered as potential competing interests: M.B.G. has received funding grants from Skeletal Dynamics, Acumed, Arthrex, Stryker, and Konica Minolta; is a board member of the American Society for Surgery of the Hand, and is an associate editor for the *Journal of Hand Surgery* and *Surgical Techniques in Orthopedics*. He had no involvement in the peer review of this article and had no access to information regarding its peer review. All other authors (J.G., S.B.C., A.L.G., N.S., E.R.W.) declare that they have no known competing financial interests or personal relationships that could have appeared to influence the work reported in this paper.

## Supplementary Data

Video 1 Video of an arthroscopic assisted distal radial ulnar joint reconstruction using a modified anatomic ligament reconstruction. This technique utilizes a palmaris longus graft passed through the ulnar fovea to a radial bone tunnel at the level of the distal radioulnar joint and then shuttled out the ulnar diaphysis. It is assisted by arthroscopy to assess the integrity of the triangular fibrocartilage complex and aid in debridement, graft passage, and tensioning.
